# Safety assessment of anticoagulation therapy in patients with hemorrhagic cerebral venous thrombosis

**Published:** 2013

**Authors:** Kavian Ghandehari, Hamid Reza Riasi, Ali Noureddine, Shahram Masoudinezhad, Siamak Yazdani, Mohammad Mousavi Mirzae, Atena Sharifi Razavi, Kosar Ghandehari

**Affiliations:** 1 Professor, Department of Cerebrovascular Disease AND Neurocognitive Research Center, School of Medicine, Mashhad University of Medical Sciences, Mashhad, Iran; 2 Assistant Professor, Department of Neurology, School of Medicine, Birjand University of Medical Sciences, Birjand, Iran; 3 Resident, Department of Neurology, School of Medicine, Mashhad University of Medical Sciences, Mashhad, Iran; 4 Researcher, Neurocognitive Research Center, Mashhad University of Medical Sciences, Mashhad, Iran

**Keywords:** Cerebral Vein, Thrombus, Hemorrhagic

## Abstract

**Background:**

Anticoagulation therapy is a routine treatment in patients with hemorrhagic cerebral venous thrombosis (CVT). However, fear of hemorrhagic complications and deterioration course following anticoagulation often disturbs the responsible physician.

**Methods:**

This was a Prospective observational study on consecutive CVT patients with hemorrhagic venous infarction or subarachnoid hemorrhage (SAH) admitted in Ghaem Hospital, Mashhad, Iran, during 2006-2012. The diagnosis of CVT in suspected cases was confirmed by magnetic resonance imaging/magnetic resonance venography (MRI/MRV), and computerized tomography (CT) angiography following established diagnostic criteria. Demographic data, clinical manifestations from onset to end of the observation period, location of thrombus, location and size of infarction and hemorrhage, and clinical course during treatment were recorded. Choice of the treatment was left to the opinion of the treating physician. Clinical course during 1 week of treatment was assessed based on the baseline modified National Institute of Health Stroke Scale (NIHSS) score. Three or more points decrease or increase of modified NIHSS after 1 week of treatment was considered as improvement or deterioration courses, respectively. Other clinical courses were categorized as stabilization course.

**Results:**

102 hemorrhagic CVT patients (80 females, 22 males) with mean age of 38.6 ± 8 years were prospectively investigated. Of the 102 hemorrhagic CVT patients in the acute phase, 52 patients (50.9%) were anticoagulated with adjusted dose intravenous heparin infusion and 50 cases (49.1%) received subcutaneous enoxaparin 1mg/Kg twice daily. Decreased consciousness had a significant effect on the clinical course of the patients (X^2^ = 9.493, df = 2, P = 0.009). Presence of SAH had no significant effect on the clinical course of our anticoagulated hemorrhagic CVT cases (X^2^ = 0.304, df = 2, P = 0.914). Extension of Infarction in more than two thirds of a hemisphere had a significant influence on the distribution of clinical courses (X^2^ = 5.867, df = 2, P = 0.044). Difference in distribution of clinical course among the two groups of our hemorrhagic CVT patients was not significant (X^2^ = 8.14, df = 1, P = 0.87).

**Conclusion:**

Patients with hemorrhagic CVT without other contraindication for anticoagulation should be treated either with dose-adjusted intravenous heparin or body-weight-adjusted subcutaneous low molecular-weight heparin.

## Introduction

Cerebral venous thrombosis (CVT) is sometimes a diagnostic and therapeutic challenge. There are several rationales for anticoagulation therapy in CVT; to prevent thrombus growth, to facilitate recanalization, and to prevent deep vein thrombosis. Cerebral venous infarction with hemorrhagic transformation or intracerebral hemorrhage is commonly present at the time of diagnosis of CVT and it may complicate treatment.^1^ One third of patients with CVT present with intracerebral hemorrhage or hemorrhagic venous infarcts. Hemorrhage on CT is among predictors of bad outcome of CVT patients.^2^ Isolated subarachnoid hemorrhage (SAH) may also occur rarely due to CVT. 0.8% of patients in an international study of cerebral vein and dural sinus thrombosis had isolated SAH.^3^ Other studies suggested low rates of cerebral hemorrhage after anticoagulation for CVT.^1^ In the special situation of CVT with cerebral hemorrhage on presentation, the clinician must balance the risks and benefits of anticoagulation, and low-intensity anticoagulation may be considered if possible in favor of no anticoagulation until such time as it might be safe to use full intensity anticoagulation.^1, 2^ Although intracerebral hemorrhage and SAH are sometimes found in patients with CVT and lead to worse outcome, predictors of outcome in this subgroup of CVT patients are only evaluated in a few studies.^1–3^ This subgroup of CVT patients can be the target of new therapeutic strategies. The aim of this study was the identification of predictors of outcome and observation of clinical course of patients with hemorrhagic CVT who underwent anticoagulation in their acute phase.

## Materials and Methods

Consecutive CVT patients with hemorrhagic venous infarction or SAH admitted in Ghaem Hospital, Mashhad, Iran, enrolled into a prospective observational study during 2006-2012. The diagnosis of CVT in suspected cases was confirmed by magnetic resonance Imaging/ magnetic resonance venography (MRI/MRV), computerized tomography (CT) angiography, and catheter angiography following established diagnostic criteria.^1, 4^ Venous thrombus frequently appears as isointense on T_1_ and hypointense on T_2_ weighted images in the first week. By the second week it appears as hyperintense on both T_1_ and T_2_ weighted images.^1, 4^

Absence of flow void with alternation of signal intensity in the dural sinus is the principal early sign of CVT in MRI.^1, 4^ MRV was done in all of the cases to define the extent of CVT in MRI positive cases and to rule out CVT in MRI negative cases.^1^ Cerebral angiography and CT angiography was done in selected situations in which MRI/MRV results are inconclusive.^1^ Patients with high clinical suspicion of CVT and negative MRI/MRV results underwent serial MRI/MRV.^1^ Demographic data, clinical manifestations from onset to end of observation period, location of thrombus, location and size of infarction and hemorrhage, imaging method used, and clinical course during treatment were recorded.^3, 5^ Early hemorrhage was defined as any hemorrhagic transformation of venous infarction present on CT or MRI at time of diagnosis. Delayed hemorrhage was defined as any hemorrhagic transformation of venous infarction or SAH that was not present on CT or MRI scan at time of diagnosis but occurred later, or later a new hemorrhage occurred elsewhere.^5^

The choice of the treatment was left to the opinion of the treating physician.^5^ All of the CVT patients with massive hemorrhage or extensive venous infarction received mannitol 20% serum in order to decrease intracranial pressure. Decompressive craniotomy was also performed in these cases according to the neurosurgeon's decision. This therapeutic approach for decreasing intracranial pressure was performed regardless of the choice of anticoagulation method. CVT patients in our institution were routinely administered an adjusted dose of heparin with continuous intravenous infusion to achieve an activated partial thromboplastin time twice the pretreatment value during 2006-2009. Two thirds of these patients received subcutaneous enoxaparin 1mg/Kg twice daily during 2009-2012. The clinical course during 1 week of treatment was assessed based on the modified National Institute of Health Stroke Scale (NIHSS) score.^6^ Three or more points decrease or increase in modified NIHSS score after 1 week of treatment was considered as improvement or deterioration courses, respectively. Other clinical courses were categorized as stabilization. The clinical course of hemorrhagic CVT patients who received other anticoagulation methods was not included in this article due to their low number and bias prevention. Outline of the venous infarction was carefully determined in the brain MRI by using a point grid. Subsequently, the infarct surface area was manually calculated, based on square millimeter.^7, 8^ Size of venous infarctions was categorized as ≥ 1/3 and ≥ 2/3 of cerebral hemisphere.

The extent of hemorrhagic transformation within venous infarction was also categorized as ≥ 1/3 and ≥ 2/3 of infarct area. All of the CVT patients underwent a standard battery of diagnostic investigations for determination of the cause of their CVT.^1, 4^ Data on etiology of CVT in the east of Iran is presented elsewhere.^9^ Fisher's exact, chi-square and Student's t-test served for statistical analysis. An informed consent was signed by every patient or his/her first degree relatives, and the research was approved by our local ethics committee.

## Results

102 hemorrhagic CVT patients (80 females, 22 males) with mean age of 38.6 ± 8 years were prospectively investigated. Of the 102 hemorrhagic CVT patients in the acute phase 52 patients (50.9%) were anticoagulated with adjusted dose of intravenous heparin infusion, and 50 patients (49.1%) received weight adjusted subcutaneous enoxaparin in therapeutic dose. Additional treatments included antiepileptic drugs (48.1%), acetazolamide (90.4%), steroid (76.9%), diuretics (90.4%), and decompressive craniotomy (10%). Decreased consciousness, headache, seizure, weakness, and aphasia were found in 55.8%, 96.2%, 48.1%, 32.7%, and 7.7% of the hemorrhagic CVT patients, respectively. Of 102 hemorrhagic CVT cases 83 patients had isolated hemorrhagic venous infarction, 5 cases had isolated focal venous SAH, and 14 had both hemorrhagic venous infarction and SAH. Brain CT, MRI, and MRV were done in all of the CVT cases. CT angiography and catheter angiography were requested in 3.8% and 3.8% of the patients, respectively. The diagnosis of CVT was established by MRI/MRV in 92.3%, by CT angiography in 1.9%, and by serial MRI/MRV in 5.8% of cases.

Decreased consciousness had a significant effect on the clinical course of the patients; X^2^ = 9.493, df = 2, P = 0.009. The influence of headache (X^2^ = 5.581, df = 2, P = 0.053), seizure (X^2^ = 0.041, df = 2, P = 1), weakness (X^2^ = 0.985, df = 2, P = 0.614), and aphasia (X^2^ = 0.844, df = 2, P = 1) in the clinical course of these patients were not significant. The effect of gender on clinical course of these patients was not significant (X^2^ = 2.020, df = 2, P = 0.347). Early hemorrhage constituted 92.3% of hemorrhagic CVT in our series. Patients with early pretreatment hemorrhage had a significantly better clinical course than patients with late hemorrhage (X^2^ = 7.604, df = 2, P = 0.036. Presence of SAH had no significant effect on clinical course of our hemorrhagic CVT cases (X^2^ = 0.304, df = 2, P = 0.914). Table 1 demonstrates the statistical relation of imaging characteristics with clinical course of 102 anticoagulated hemorrhagic CVT cases. Among the 83 patients with isolated hemorrhagic venous infarction 91.4% had infarct in the anterior circulation, 4.3% had infarct in posterior circulation, and 2.1% had mixed infarcts. Table 2 illustrates the distribution of clinical course categories in the two therapeutic groups of our 102 hemorrhagic CVT patients. Difference in distribution of clinical course categories among the two groups of our hemorrhagic CVT patients who received intravenous heparinization and subcutaneous enoxaparin was not significant; X^2^ = 8.14, df = 1, P = 0.87.

**Table 1:** Relation of imaging characteristics with clinical course of 102 anticoagulated hemorrhagic CVT cases

Imaging Characteristic\ Clinical course	X2	df	p
Infarction ≥ 1/3 hemisphere*	4.63	2	0.09
Infarction ≥ 2/3 hemisphere*	5.87	2	0.04
Hemorrhagic transformation ≥ 1/3 of infarct area*	4.62	2	0.14
Hemorrhagic transformation ≥ 2/3 of infarct area*	3.44	2	0.14
Superior sagital sinus thrombus**	0.02	2	1.00
Left transverse sinus thrombus**	1.36	2	0.63
Right transverse sinus thrombus**	1.79	2	0.48
Straight sinus thrombus**	2.32	2	0.29
Multiple sinus thrombus**	0.64	2	0.81

* Within 83 patients with isolated hemorrhagic venous infarction

** Within 102 patients with hemorrhagic venous infarction or venous SAH

**Table 2:** Distribution of clinical course categories in two therapeutic groups of our hemorrhagic CVT patients

Therapeutic group\ clinical course	Improvement Number of treated cases	Stabilization Number of treated cases	Deterioration Number of treated cases
Adjusted dose intravenous infusion of heparin (52)	36	10	6
Weight adjusted subcutaneous Enoxaprin (50)	35	10	5
Total (102)	71	20	11

## Discussion

Deterioration in clinical course occurred only in 11% of our hemorrhagic CVT patients who received anticoagulation therapy, which confirms the high level of safety of this treatment in these cases.

Observational studies reported the range of risks for Intracerebral hemorrhage (ICH) after anticoagulation for CVT to be from 0 to 5.4%.^1^ The outcome of CVT remains largely unpredictable. It is not unusual to see deeply comatose or severely hemiplegic patients recover dramatically without any sequelae.^2^ Conversely, a patient with headache as the only presenting symptom can suddenly worsen, with a dense hemiplegia if thrombus spreads from a sinus to a cerebral vein.^2^ Interestingly, it is well established that clinical recovery starts much more rapidly than vessel recanalization and can occur even in the absence of recanalization.^2^ Heparin has been widely used in hemorrhagic CVT as evidence has accumulated that it is both effective and safe.^1, 2^ Our study population is not representative of all CVT patients, but consists of a subgroup of patients who have a higher risk for poor outcome when compared with CVT patients without hemorrhage. This selective evaluation of hemorrhagic CVT patients is an advantage of our study. The largest study performed is an international study on cerebral vein and dural sinus thrombosis, which included 624 patients in 21 countries.^3^ Nearly all of the patients were treated with anticoagulation initially and mortality was 8.3%.^3^ 39% of these 624 patients had an early hemorrhagic transformation of venous infarction or SAH.^3^ Patients with early hemorrhage in this study had 6% mortality rate within 1 month and 21% rate of death or dependency at month 6.^5^

Among the CVT patients with early hemorrhage in this study, independent predictors of death or dependency at 6 months were old age, male gender, having a thrombus of the deep cerebral venous system, and having a motor deficit.^5^ Decreased consciousness and extension of Infarction in more than two thirds of a hemisphere had a significant effect on the clinical course of patients in our study. Of those with delayed hemorrhagic complication who had enrolled in the international study of CVT, those who had a worse outcome were less likely to have been treated by heparin.^5^ While, our anticoagulated CVT patients with early pretreatment hemorrhage had a significantly better clinical course than patients with late hemorrhage. A placebo-controlled trial of 20 patients with CVT assessed intravenous unfractionated heparin used dose adjustment to achieve an activated partial thromboplastin time twice the pretreatment value.^10^ Two patients treated with placebo and one patient treated with heparin developed ICH. This clinical trial was stopped after 20 patients because of the dramatic difference in death and dependency observed between the two groups.^10^ Heparin therapy leads to a 14% absolute risk reduction in mortality, and 15% in death or dependency, with relative risk reductions of 70% and 56%, respectively.^2^ One other trial of 59 patients compared subcutaneous nadroparin dosed on the basis of body weight with placebo.^11^ CVT patients significantly benefited from anticoagulation therapy and there was no symptomatic ICH due to anticoagulation in patients of both trials.^11^ No worsening attributable to new or enlarged cerebral hemorrhage was observed in CVT patients who received nadroparin, even among those who had hemorrhagic lesions demonstrated on CT images.^11^

In the nadroparin trial, patients with isolated intracranial hypertension had a better outcome than those with seizures, focal deficits, or coma. Nadroparin caused an absolute risk reduction of 7% and a relative risk reduction of 38% for poor outcomes defined as death or a Barthel score of < 15.^11^ In the special situation of CVT with cerebral hemorrhage on presentation, even in the absence of anticoagulation, hemorrhage is associated with adverse outcome and mortality.^1, 12^ However, all 6 deaths in the nadroparin trial occurred in the group of 29 patients with hemorrhage on their pretreatment CT scan.^2, 11^ These 29 cases were equally divided between treatment groups, and none of the deaths were attributed to new or enlarged hemorrhage during treatment.^2, 11^

In a retrospective study of 102 patients with CVT, 43 had an ICH.^1^ Among the 27 patients who were treated with dose-adjusted intravenous heparin after ICH, 15% died and 52% recovered completely.^1^ Our observational study has not shown a significant difference in the clinical course of our hemorrhagic CVT patients who received either a therapeutic dose of intravenous heparin or subcutaneous enoxaparin. A recent prospective, cohort study compared 119 CVT patients receiving low-molecular weight heparin and 302 who received unfractionated heparin.^13^ Significantly more patients treated with low-molecular weight heparin were functionally independent after 6 months.^13^ This nonrandomized study suggested a better efficacy and safety for low-molecular weight heparin in comparison to unfractionated heparin in CVT patients.^13^ However, the use of intravenous heparin is particularly recommend in critically ill patients; because the activated partial thromboplastin time may normalize within 1 hour after discontinuation of the infusion if complications occur, or surgical intervention or repeated lumbar punctures are necessary.^12^ In general, limited data from randomized controlled clinical trials in combination with observational data on outcomes and bleeding complications of anticoagulation support a role for anticoagulation in treatment of CVT, regardless of the presence of pretreatment ICH, and that anticoagulation appears safe and effective.^1, 2^

## Conclusion

Our observational therapeutic study showed the safety of anticoagulation therapy and favorable clinical course in patients with hemorrhagic venous infarction or venous SAH. Patients with hemorrhagic CVT without other contraindication for anticoagulation should be treated either with dose-adjusted intravenous heparin or body-weight-adjusted subcutaneous low-molecular weight heparin.
